# Probiotics Fermented Bitter Melon Juice as Promising Complementary Agent for Diabetes Type 2: Study on Animal Model

**DOI:** 10.1155/2020/6369873

**Published:** 2020-02-28

**Authors:** Laksmi Hartajanie, S. Fatimah-Muis, K. Heri-Nugroho HS, Ign Riwanto, M. Sulchan

**Affiliations:** ^1^Diponegoro University Faculty of Medicine, Semarang, Indonesia; ^2^Soegijapranata Catholic University Faculty of Food Technology, Semarang, Indonesia

## Abstract

*Background and Aim*. Bitter melon (*Momordica charantia*/MC) contains charantin that has antidiabetic properties as an *α*-glucosidase inhibitor and antioxidative properties. Lactic acid fermentation using *Lactobacillus fermentum* LLB3 increased its antioxidative properties. The study was aimed to analyse the difference of the treatment that influences blood glucose and SOD level before and after treatment compared to acarbose. *Experimental procedure*. A total of 24 male Sprague-Dawley rats were used. Diabetes type 2 was induced by a single dose (60 mg/kg) of streptozotocin (STZ) and 120 mg/kg of nicotinamide, intraperitoneally. Following three days of STZ induction, the animals were randomly divided into four groups. Groups 1, 2, 3, and 4 were given acarbose 40 mg/100 g feed, MC 10 ml/kg body weight, fermented MC 10 ml/kg body weight, and distilled water, respectively, for 28 days. Glucose and SOD values were measured by spectrophotometer and ELISA, respectively. The difference between pretest and posttest data was analysed using the pair *t*-test. Data were analysed using ANOVA and Tukey HSD for post hoc analysis. Level of significance was set at 0.05.

**Results:**

Fasting glucose and postprandial blood glucose were significantly decreased in groups given MC and fermented MC but not as low as those in the acarbose group (*p* < 0.001). The value of SOD significantly increased in groups given MC and fermented MC but not as high as those in the acarbose groups (*p* < 0.001). The value of SOD significantly increased in groups given MC and fermented MC but not as high as those in the acarbose groups (

**Conclusion:**

Although MC gave significant results in increasing SOD and lowering fasting as well as postprandial blood glucose, fermented MC was better than MC, and acarbose still gave the best results.

## 1. Introduction

Diabetes mellitus is a metabolic disorder characterised by hyperglycemia, resulting from either insulin resistance or insufficient insulin release or both [1–3]. Diabetes is classified into four main groups: type I (insulin dependent), type II (non-insulin dependent), gestational diabetes, and other specific types. Type II represents 90% of diabetes cases around the world [1, 4].

Unmanaged hyperglycemia will cause microvascular and macrovascular complications. Based on Indonesian DiabCare 2008, unmanaged diabetes caused eye complications (26.4%), diabetic nephropathy (8.2%), diabetic ulcers (6.8%), cardiovascular diseases (22.6%), and other complications (8.6%) [5]. Diabetes mellitus caused 1.5 million deaths in the world in 2012 and became a major global health problem related to the projected increase in prevalence from 415 million in 2015 to 642 million in 2040 [2, 3]. Diabetes has become the third leading cause of death after cancer [4]. In Indonesia, the prevalence of diabetes mellitus was increased from 1.1 percent in 2007 to 2.1 percent in 2013 [6].

Nowadays, the management of diabetes has become a global issue, and effective treatment is needed to be found. Medical treatment for diabetes such as insulin injections and oral hypoglycemic agents caused adverse side effects such as liver problems, lactic acidosis, and gastrointestinal problem [7–10]. Therefore, there is a strong interest in searching for complementary drugs. Plants with antidiabetic properties could be used as a complementary medication, and their functional properties could be increased by fermentation [11].

Bitter melon (*Momordica charantia*) is known to be effective in control blood sugar levels [12, 13]. It contained antidiabetic phytomedicines, including charantin, insulin-like peptides, and alkaloids. These compounds play a role in increasing tolerance to glucose without increasing blood insulin levels [13–16] and stimulating adenosine monophosphate-activated protein kinase (AMPK) [16, 17]. The regulation of AMPK is believed to improve energy metabolism in metabolic syndrome [18]. Probiotics administration can reduce the risk of developing type 2 diabetes in prediabetes patients [19, 20]. Probiotic consumption of 10^6^–10^8^ CFU per day for eight weeks can increase glucose metabolism in the modest degree [20] and significantly increase the activity of superoxide dismutase (SOD) [19].

Fermented vegetables have better functionality than unfermented ones [21, 22]. *Momordica charantia* (MC) juice fermented using *Lactobacillus fermentum* LLB3 has increased antioxidant activity by 15% [11]. Side effects of administration of bitter melon or probiotics in preclinical and clinical trials are not yet to be found [19, 20, 23–34]. This study was carried out because, until now, there has been no preclinical trial to find out the effectivity of fermented bitter melon juice in managing glycemic status.

## 2. Materials and Methods

### 2.1. Preparation of Bitter Melon Juice

Fresh unripe bitter melon was purchased from Hortimart plantation. Bitter melon was picked 40 days after planting with an average weight of 200 g. The fruit was washed and split to remove the seeds. The flesh was extracted into juice without adding water. The juice was pasteurised at 70°C for 5 minutes. *Lactobacillus fermentum* LLB3 was fermented in MRSB and incubated at 37°C for 24 hours (OD_600_ = 1). Twenty ml inoculum inoculated aseptically into 180 ml pasteurised juice and fermented at 37°C for 24 hours.

### 2.2. Animals and Treatments

The animal's treatments were followed by the methods of Abdellatief et al. [35]. A total of 24 male Sprague-Dawley rats at eight weeks and weighing 170–200 g were used for the study. They were obtained from House of Experimental Rats CNFS, Gadjah Mada University, Yogyakarta, Indonesia. Environmental conditions such as 12 : 12 hours of light/dark cycle, the ambient temperature of 25 ± 1°C, normal humidity, and proper sanitation were maintained in order to minimise stress during the experiment. The rats were housed in individual stainless steel cages. They were fed on a standard diet of chow and were given unrestricted access to water. The rats were acclimatised for seven days before initiation of the experiment. Animal facilities, their management, and handling during the experiment were done in compliance with the Guidelines for Care and Use of Laboratory Animals of CNFS Gadjah Mada University and were approved by the Research Ethics Committee of the Faculty of Medicine, Diponegoro University number 98/EC/H/FK-RSDK/VII/2018.

### 2.3. Induction of Type 2 Diabetes

Overnight fasting rats have injected intraperitoneally a single dose (60 mg/kg BW) of STZ, which freshly dissolved in 0.1 M citrate buffer (pH = 4.5). The rats have injected 120 mg/kg BB of nicotinamide dissolved in normal saline 15 min before STZ injection. Type 2 DM was determined by fasting blood glucose over 200 mg/dL on the third day after induction [36].

### 2.4. Experimental Design

After 72 h injection of STZ, rats were distributed into four groups (*n* = 6). Acarbose group (DM-Ac) rats were given 40 mg/100 g feed. Bitter melon group (DM-MC) rats were given 10 mg/kg BW bitter melon juice orally. Fermented bitter melon juice was prepared, according to Hartajanie et al. (2018) [11]. Fermented bitter melon group (DM-PMC) rats were given 10 mg/kg BW fermented bitter melon juice containing 10^7^ probiotics orally. Control group (DM-Ctrl) rats were given only distilled water. The rats were treated daily for a period of 28 days.

### 2.5. Collection of Samples

Before and after the trial period, the overnight fasting rats were profoundly anaesthetised, and blood samples were collected from vena sinus orbitalis [37, 38] and centrifuged at 3000 rpm for 15 min to obtain serum, which were used for estimation of glucose and SOD.

### 2.6. Evaluation of Fasting Blood Glucose and Postprandial Blood Glucose

Fasting blood glucose and postprandial blood glucose were measured by the commercial kits purchased from DiaSys [39].

### 2.7. Evaluation of Serum Oxidative Stress

Activities of superoxide dismutase (SOD) were measured by the commercial kits purchased from BioVision [40].

### 2.8. Statistical Analysis

Sampling was conducted before (pretest) and after (posttest) treatments. The results were reported as mean ± standard deviation (*n* = 6). As all data were normally distributed, the significance of differences before and after treatments was determined using the pair *t*-test. The significance of differences between the groups was determined using one-way analysis of variance (ANOVA), calculated by SPSS version 20 program, with a significance level of *p* < 0.05 by the Tukey HSD Test.

## 3. Results

**Figure 1:**
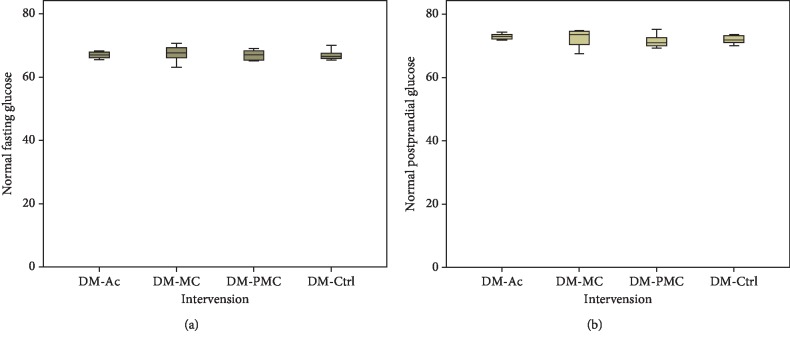
Baseline normal blood glucose: (a) fasting blood glucose; (b) postprandial blood glucose.

Baseline fasting and postprandial blood glucose are shown in Figure 1. In each group, blood glucose levels were not significantly different, and their value ranged from 62.1 to 67.4 mg/dl (fasting blood glucose) and from 71.5 to 72.9 mg/dl (postprandial blood glucose).

### 3.1. Antidiabetic Effect of Fermented Bitter Melon Juice

**Figure 2:**
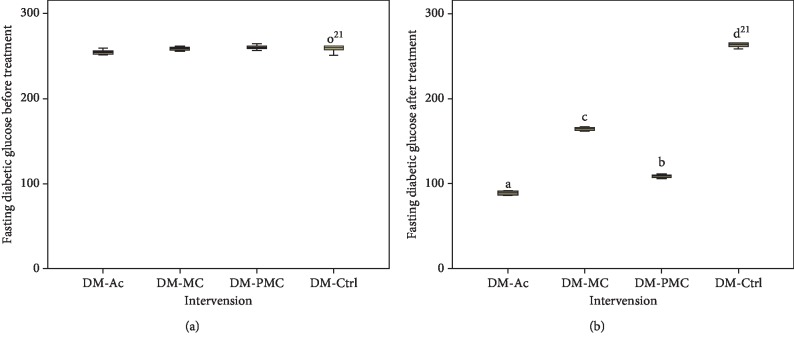
Fasting diabetic glucose: (a) before treatment; (b) after treatment.

**Figure 3:**
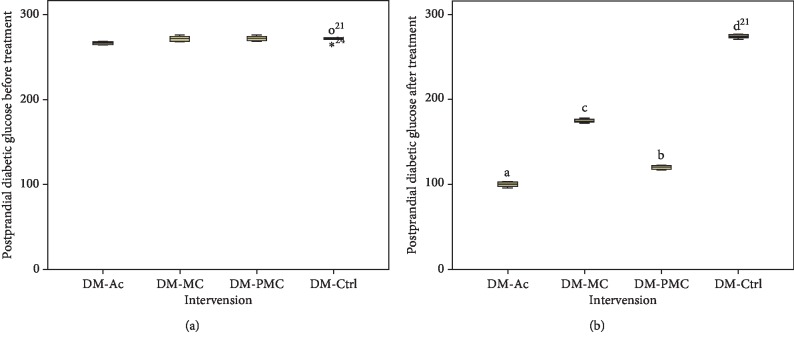
Postprandial diabetic glucose: (a) before treatment; (b) after treatment.

The administration of fermented and nonfermented bitter melon juice caused a significant decrease in the levels of blood glucose (Figures 2 and 3). Blood glucose levels significantly decreased (*p* < 0.05) after treatment with bitter melon juice (DM-MC) and fermented bitter melon juice (DM-PMC) when compared to the diabetic control group (DM-Ctrl). Overall, DM-PMC was better than DM-MC.

### 3.2. Effect on Oxidative Status

**Figure 4:**
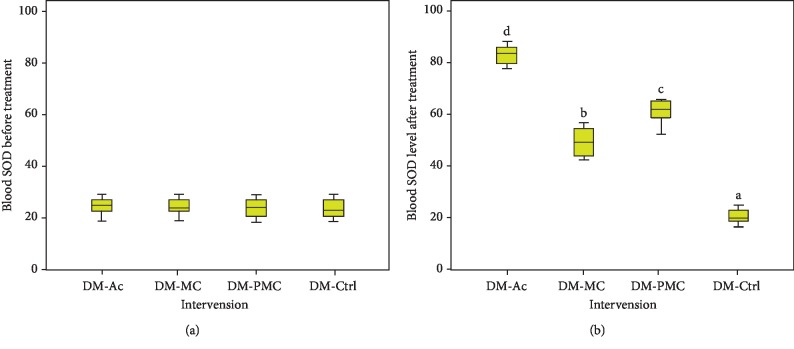
SOD level: (a) before treatment; (b) after treatment.

There were significant changes in the concentrations of SOD (Figure 4). After administration nonfermented and fermented bitter melon juice, a significant increase (*p* < 0.05) in SOD concentrations was observed in comparison with diabetic control (DM-Ctrl)). In line with the hypoglycemic effect, DM-PMC was better than DM-MC.

### 3.3. Correlation between Blood Glucose and SOD

Correlation between fasting blood glucose and SOD was *r* = −0.966, *p*=0.001, while between SOD and postprandial blood glucose was *r* = −0.967, *p*=0.001.

## 4. Discussion

Groups were comparable regarding baseline fasting and postprandial glucose level and also SOD level; therefore, to evaluate the efficacy of bitter melon and fermented bitter melon, it was enough to compare the data from the posttest only.

Treatment of diabetic rats with fermented MC juice resulted in a significant decrease in fasting and postprandial blood glucose in comparison with nonfermented one, although the decrease was inferior compared to acarbose (Figures 2 and 3). This study revealed that fermented MC juice has a more powerful antihyperglycemic effect than nonfermented one. However, acarbose showed a stronger effect than MC and fermented MC juice. The better antidiabetic potential can be attributed to the higher content of antioxidants in fermented MC juice.

Bitter melon contains active components (charantin, triterpenoid, and *p*-polypeptides) that manage blood sugar level [1, 41]. Charantin acts as an *α*-glucosidase inhibitor. Triterpenoids act as insulin sensitisers, and *p*-polypeptides act as insulin-mimetic, which can increase cellular glucose uptake and decrease insulin resistance [1, 24, 41]. Although there was a significant reduction, the administration of MC juice regarding glucose levels was inferior compared to the fermented one. Reducing glucose levels in the fermented group is associated with charantin, which works as an antioxidant and *α*-glucosidase inhibitor. Fermenting bitter melon juice using *Lactobacillus fermentum* LLB3 increased antioxidant activity by 15%, and there is an increase in the inhibitory activity of *α*-glucosidase in vitro [11, 12].

The stronger effect of fermented MC on reducing blood glucose can be explained by the additional amount of *α*-glucosidase produced by lactic acid bacteria in fermented MC. Lactic acid bacteria produce the enzyme *β*-glucosidase, which hydrolyses charantin to sitosteryl glucoside and glucoside stigmasteryl that caused an increase in inhibition of *α*-glucosidase activity [12, 14]. Inhibition of *α*-glucosidase causes a decrease in carbohydrate absorption and prevents postprandial spike [42]. The presence of probiotic bacteria in fermented bitter melon juice plays an essential role in decreasing fasting and postprandial glucose levels. Probiotic bacteria produce peptides that play a role in glucose absorption through PI3-K pathways [43]. Probiotic bacteria also produce SCFA, which has the role of increasing host cell epithelial function [44].

The administration of nonfermented and fermented MC juice has been shown to increase SOD levels (Figure 4). There was a significant increase in SOD levels in subjects administered with fermented MC juice in comparison to nonfermented one. Although the increase is inferior to acarbose, fermented MC administration provided a significant improvement in the antioxidant status. High glucose levels stimulate the formation of free radicals. Antioxidants in MC juice and fermented MC juice scavenging free radicals produced from glucose oxidation resulted in increasing SOD levels. In the intestines, probiotic bacteria produce bacteriocins, bile salt hydrolase (BSH), short-chain fatty acids (SCFAs), and peptides. SCFA acts as an antioxidant so that the presence of probiotics is in line with the increase in the antioxidant status of the subject. Peptides reduce blood sugar level through the PI3-K pathway [43].

**Figure 5:**
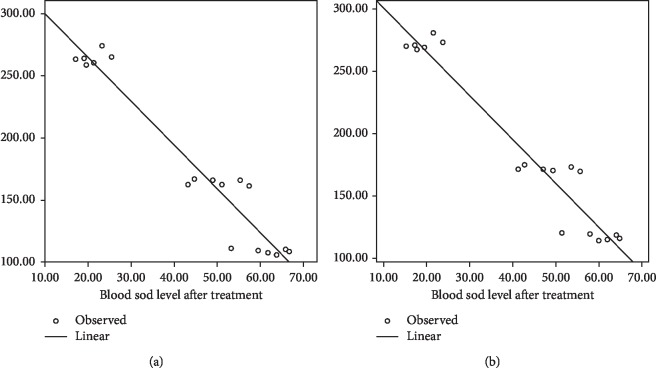
Correlation of (a) SOD vs. fasting diabetic glucose and (b) SOD vs. postprandial diabetic glucose.

SOD plays a role in catalysing the dismutation of superoxide anion (O_2_^−^) into hydrogen peroxide and molecular oxygen [45, 46]. There was a robust negative correlation between both fasting and postprandial blood glucose and SOD levels (Figure 5). MC juice contained antioxidants, and fermentation increased its antioxidant activity. Antioxidant intake can overcome oxidative stress, reduce ROS, and increase antioxidant enzymes, which has been shown to prevent diabetes mellitus [47]. Bioactive components of bitter melon can control blood sugar because it has a protective and regenerative effect on *β* cells and increases insulin sensitivity and *α*-glucosidase inhibitors [23, 26].

Although the antihyperglycemic effect is still below the acarbose potency, fermented MC juice has a more powerful antihyperglycemic effect than the nonfermented one. This effect can be attributed to the higher content of antioxidants in fermented MC juice. Further studies are required to determine the effect of probiotics in fermented MC juice on the improvement of pancreatic *β* cell structure.

## Data Availability

The data used to support the findings of this study are included within the article and supplementary information file.

## Supplementary Materials

Supplementary Materials Table 1 lists the baseline of glucose level before induction. Table 2 lists the glucose level before-after treatment and pair *t*-test. Table 3 lists the SOD level before-after treatment and pair *t*-test. Table 4 lists the correlation between glucose and SOD pair *t*-test. Statistical analyses comprise ^∗^normality, ^∗^homogeneity, ^∗^ANOVA, and ^∗^pair *t*-test. FBG = fasting blood glucose; PPBG = postprandial blood glucose. Treatment 1 = DM rats + acarbose; treatment 2 = DM rats + bitter melon juice; treatment 3 = DM rats + fermented bitter melon juice; treatment 4 = DM rats + distilled water.
