# Supplementary material for: Probiotics Fermented Bitter Melon Juice as Promising Complementary Agent for Diabetes Type 2: Study on Animal Model
Source: J Nutr Metab. 2020 Feb 28;2020:6369873. doi: 10.1155/2020/6369873 (PMC7064845; doi:10.1155/2020/6369873)
Supplement: Supplementary Materials — Table 1 lists the baseline of glucose level before induction. Table 2 lists the glucose level before-after treatment and pair t-test. Table 3 lists the SOD level before-after treatment and pair t-test. Table 4 lists the correlation between glucose and SOD pair t-test. Statistical analyses comprise ∗normality, ∗homogeneity, ∗ANOVA, and ∗pair t-test. FBG = fasting blood glucose; PPBG = postprandial blood glucose. Treatment 1 = DM rats + acarbose; treatment 2 = DM rats + bitter melon juice; treatment 3 = DM rats + fermented bitter melon juice; treatment 4 = DM rats + distilled water. [file 6369873.f1.pdf]

Table 1. Baseline normal blood glucose

Characteristics	DM-Ac	DM-MC	DM-PMC	DM-Ctrl
Fasting blood glucose	62.1 ±1.09	67.4 ±2.66	67.0 ±1.60	67.0 ±1.67
Postprandial blood glucose	72.9±0.89	72.4 ±2.89	71.5 ±2.13	71.9 ±1.32

Values are represented as Mean ± SD (n = 6). Means within the same row carrying different superscripts are significant at $p < 0.05$.

Table 2. Within-group and between-group comparisons of blood glucose before and after administration

Groups	*pretest*	*posttest*	p^{a}
Fasting Blood Glucose			
1 (DM-Ac)	255.0 ± 2.79	88.9 ± 2.16^{a}	0.000*
2 (DM-MC)	259.0 ± 2.22	164.3 ± 2.21^{c}	0.000*
3 (DM-PMC)	260.4 ± 2.80	109.1 ± 2.07^{b}	0.000*
4 (water)	259.7 ± 5.52	264.4 ± 5.22^{d}	0.007*
F *test*	2.802	3600.286	
P	0.066	0.000	
Post Prandial Blood Glucose			
1 (DM-Ac)	266.3 ± 1.81^{a}	100.4 ± 2.90 ^{a}	0.000*
2 (DM-MC)	271.4 ± 3.15^{ab}	174.9 ± 2.04^{c}	0.000*
3 (DM-PMC)	271.8 ± 2.86^{b}	120.1 ± 2.49^{b}	0.000*
4 (water)	271.6 ± 4.89^{ab}	275.3 ± 4.73^{d}	0.014*
F *test*	3.702	3573.526	
P	0.059	0.000	

Values are represented as Mean ± SD (n = 6). Means within the same column carrying different superscripts are significant at $p < 0.05$. [a]Obtained from a paired t-test for the pretest and posttest comparisons ($p < 0.05$). Grup DM-Ac rats were given acarbose 40 mg/100 g feed. Group DM-MC rats were given 10 mg/kg BW bitter melon juice orally. Group DM-PMC rats were given 10 mg/kg BW fermented bitter melon juice containing 10^7 probiotics orally. Group DM-Ctrl rats were given only distilled water. The rats were treated daily for a period of 28 days.

Table3. Within-group and between-group comparisons of oxidative marker (SOD) before and after administration

Groups	*pretest*	*posttest*	p^a
1 (DM-Ac)	25.2 ± 3.66 [a]	84.3 ± 3.95[e]	0.000*
2 (DM-MC)	24.9 ± 3.59[a]	50.1 ± 5.68[b]	0.000*
3 (DM-PMC)	24.5 ± 3.97 [a]	61.8 ± 4.98[c]	0.000*
4 (water)	24.2 ± 4.15 [a]	21.0 ± 3.09[a]	0.008*
F *test*	0.080	202.510	
P	0.970	0.000	

Values are represented as Mean ± SD (n = 6). Means within the same column carrying different superscripts are significant at p < 0.05. [a]Obtained from a paired t test for the pretest and posttest comparisons (p < 0.05).[b]the difference between pretest and posttest. Grup DM-Ac rats were given acarbose 40 mg/100 g feed. Group DM-MC rats were given 10 mg/kg BW bitter melon juice orally. Group DM-PMC rats were given 10 mg/kg BW fermented bitter melon juice containing 10^7 probiotics orally. Group DM-Ctrl rats were given only distilled water. The rats were treated daily for a period of 28 days.

Table 4. Coefficient correlation between blood glucose vs SOD

	FBG	PPBG	SOD
FBG	1	1,000**	-0,943**
PPBG	1,000**	1	-0,941**
SOD	-0,943**	-0,941**	1

**highly significant correlation (p < 0,01) based on Pearson correlation

* significant correlation (p < 0,05) based on Pearson correlation

Pretest

Normality

Tests of Normality

	Kolmogorov-Smirnov[a]			Shapiro-Wilk		
	Statistic	df	Sig.	Statistic	df	Sig.
FBG_Pre	,108	24	,200*	,969	24	,642
PPBG_Pre	,086	24	,200*	,978	24	,858
SOD_Pre	,130	24	,200*	,922	24	,064

*. This is a lower bound of the true significance.

a. Lilliefors Significance Correction

Homogenity

Test of Homogeneity of Variance

		Levene Statistic	df1	df2	Sig.
FBG_Pre	Based on Mean	,805	3	20	,506
	Based on Median	,816	3	20	,500
	Based on Median and with adjusted df	,816	3	9,850	,514
	Based on trimmed mean	,807	3	20	,505
PPBG_Pre	Based on Mean	,561	3	20	,647
	Based on Median	,560	3	20	,648
	Based on Median and with adjusted df	,560	3	8,650	,655
	Based on trimmed mean	,561	3	20	,647
SOD_Pre	Based on Mean	,259	3	20	,854
	Based on Median	,282	3	20	,838
	Based on Median and with adjusted df	,282	3	18,546	,838
	Based on trimmed mean	,267	3	20	,848

Anova

ANOVA

		Sum of Squares	df	Mean Square	F	Sig.
FBG_Pre	Between Groups	107,311	3	35,770	2,802	,066
	Within Groups	255,305	20	12,765		
	Total	362,616	23			
PPBG_Pre	Between Groups	125,841	3	41,947	3,702	,029
	Within Groups	226,598	20	11,330		
	Total	352,439	23			
SOD_Pre	Between Groups	3,545	3	1,182	,080	,970
	Within Groups	296,449	20	14,822		
	Total	299,993	23			

Homogeneous subsets

FBG_Pre

Tukey HSD

Treatment	N	Subset for alpha = 0.05
		1
1	6	254,9587
2	6	259,0220
4	6	259,7107
3	6	260,3994
Sig.		,069

Means for groups in homogeneous subsets are displayed.

a. Uses Harmonic Mean Sample Size = 6,000.

PPBG_Pre

Tukey HSD

Treatment	N	Subset for alpha = 0.05	
		1	2
1	6	266,3233	
2	6	271,4200	271,4200
4	6	271,6267	271,6267
3	6		271,7650
Sig.		,058	,998

Means for groups in homogeneous subsets are displayed.

a. Uses Harmonic Mean Sample Size = 6,000.

SOD_Pre

Tukey HSD

Treatment	N	Subset for alpha = 0.05
		1
4	6	24,1633
3	6	24,4900
2	6	24,8983
1	6	25,1700
Sig.		,968

Means for groups in homogeneous subsets are displayed.

a. Uses Harmonic Mean Sample Size = 6,000.

Posttest

Normality

	Kolmogorov-Smirnov[a]			Shapiro-Wilk		
	Statistic	df	Sig.	Statistic	df	Sig.
FBG_Post	,241	24	,001	,812	24	,000
PPBG_Post	,240	24	,001	,812	24	,000
SOD_Post	,138	24	,200*	,926	24	,081

*. This is a lower bound of the true significance. a. Lilliefors Significance Correction

Tests of Normality

	Treatment	Kolmogorov-Smirnov[a]			Shapiro-Wilk		
		Statistic	df	Sig.	Statistic	df	Sig.
FBG_Post	P1	,212	6	,200*	,880	6	,271
	P2	,262	6	,200*	,876	6	,252
	P3	,142	6	,200*	,985	6	,973
	P4	,247	6	,200*	,901	6	,383
PPBG_Post	P1	,210	6	,200*	,910	6	,435
	P2	,288	6	,130	,927	6	,557
	P3	,236	6	,200*	,868	6	,220
	P4	,256	6	,200*	,869	6	,221
SOD_Post	P1	,171	6	,200*	,973	6	,909
	P2	,163	6	,200*	,949	6	,733
	P3	,165	6	,200*	,913	6	,454
	P4	,174	6	,200*	,976	6	,929

*. This is a lower bound of the true significance.

a. Lilliefors Significance Correction

Homogenity

Test of Homogeneity of Variance

		Levene Statistic	df1	df2	Sig.
glu_puasaPost	Based on Mean	1,283	3	20	,307
	Based on Median	,980	3	20	,422
	Based on Median and with adjusted df	,980	3	6,844	,456
	Based on trimmed mean	1,134	3	20	,359
glukosa2jPost	Based on Mean	,976	3	20	,424
	Based on Median	,509	3	20	,681
	Based on Median and with adjusted df	,509	3	9,378	,686
	Based on trimmed mean	,848	3	20	,484
SOD_Post	Based on Mean	,822	3	20	,497
	Based on Median	,769	3	20	,525
	Based on Median and with adjusted df	,769	3	17,214	,527
	Based on trimmed mean	,821	3	20	,498

ANOVA

		Sum of Squares	df	Mean Square	F	Sig.
FBG_Post	Between Groups	111127,103	3	37042,368	3600,286	,000
	Within Groups	205,775	20	10,289		
	Total	111332,878	23			
PPBG_Post	Between Groups	110508,096	3	36836,032	3573,526	,000
	Within Groups	206,161	20	10,308		
	Total	110714,257	23			
SOD_Post	Between Groups	12486,040	3	4162,013	202,510	,000
	Within Groups	411,042	20	20,552		
	Total	12897,082	23			

Homogenous subsets

FBG_Post

Tukey HSD

Treatment	N	Subset for alpha = 0.05			
		1	2	3	4
1	6	88,8889			
3	6		109,0909		
2	6			164,3308	
4	6				264,3939
Sig.		1,000	1,000	1,000	1,000

Means for groups in homogeneous subsets are displayed.

a. Uses Harmonic Mean Sample Size = 6,000.

PPBG_Post

Tukey HSD

Treatment	N	Subset for alpha = 0.05			
		1	2	3	4
1	6	100,4433			
3	6		120,1383		
2	6			174,8733	
4	6				275,3150
Sig.		1,000	1,000	1,000	1,000

Means for groups in homogeneous subsets are displayed.

a. Uses Harmonic Mean Sample Size = 6,000.

SOD_Post

Tukey HSD

Treatment	N	Subset for alpha = 0.05			
		1	2	3	4
4	6	20,9983			
2	6		50,0983		
3	6			61,8200	
1	6				84,2633
Sig.		1,000	1,000	1,000	1,000

Means for groups in homogeneous subsets are displayed.

a. Uses Harmonic Mean Sample Size = 6,000.

Pair t-test

Pair t – test FBG

Paired Samples Test

		Paired Differences					t	df	Sig. (2-tailed)
		Mean	Std. Deviation	Std. Error Mean	95% Confidence Interval of the Difference				
					Lower	Upper			
Pair 1	FGB_p1_Pre - FBG_p1_Post	166,06979	2,68592	1,09652	163,25109	168,88849	151,451	5	,000
Pair 2	FBG_p2_Pre - FBG_p2_Post	94,69123	3,52503	1,43909	90,99194	98,39052	65,800	5	,000
Pair 3	FBG_p3_Pre - FBG_p3_Post	151,30854	2,02613	,82716	149,18225	153,43483	182,925	5	,000
Pair 4	FBG_p4_Pre - FGB_p4_Post	-4,68320	2,57349	1,05062	-7,38391	-1,98248	-4,458	5	,007

Pair t – test PPBG

Paired Samples Test

		Paired Differences					t	df	Sig. (2-tailed)
		Mean	Std. Deviation	Std. Error Mean	95% Confidence Interval of the Difference				
					Lower	Upper			
Pair 1	PPBG_p1_Pre – PPBG_p1_Post	165,88000	2,85183	1,16426	162,88718	168,87282	142,477	5	,000
Pair 2	PPBG_p2_Pre – PPBG_p2_Post	96,54667	3,50040	1,42903	92,87322	100,22011	67,561	5	,000
Pair 3	PPBG_p3_Pre – PPGB_p3_Post	151,62667	1,44883	,59148	150,10621	153,14712	256,350	5	,000
Pair 4	PPBG_p4_Pre – PPBG_p4_Post	-3,68833	2,42389	,98955	-6,23205	-1,14461	-3,727	5	,014

Pair t – test SOD

Paired Samples Test

		Paired Differences					t	df	Sig. (2-tailed)
		Mean	Std. Deviation	Std. Error Mean	95% Confidence Interval of the Difference				
					Lower	Upper			
Pair 1	SOD_p1_Pre - SOD_p1_Post	-59,09333	5,61668	2,29300	-64,98767	-53,19899	-25,771	5	,000
Pair 2	SOD_p2_Pre - SOD_p2_Post	-25,20000	5,44637	2,22347	-30,91561	-19,48439	-11,334	5	,000
Pair 3	SOD_p3_Pre - SOD_p3_Post	-37,33000	6,65669	2,71758	-44,31577	-30,34423	-13,736	5	,000
Pair 4	SOD_p4_Pre - SOD_p4_Post	3,16500	1,80242	,73583	1,27348	5,05652	4,301	5	,008
